# Multiparametric ultrasound examination for response assessment in breast cancer patients undergoing neoadjuvant therapy

**DOI:** 10.1038/s41598-021-82141-3

**Published:** 2021-01-28

**Authors:** K. Dobruch-Sobczak, H. Piotrzkowska-Wróblewska, Z. Klimonda, P. Karwat, K. Roszkowska-Purska, P. Clauser, P. A. T. Baltzer, J. Litniewski

**Affiliations:** 1 Ultrasound Department, Institute of Fundamental Technological Research, Polish Academy of Sciences, Pawińskiego 5B, 02-106 Warsaw, Poland; 2 Radiology Department II, Maria Sklodowska-Curie National Research Institute of Oncology, 15 Wawelska St., 02-034 Warsaw, Poland; 3 Department of Pathology, Maria Sklodowska-Curie National Research Institute of Oncology, 15 Wawelska St., 02-034 Warsaw, Poland; 4 Department of Biomedical Imaging and Image-Guided Therapy, Division of Molecular and Gender Imaging, Medical University of Vienna, Vienna, Austria

**Keywords:** Biophysics, Cancer, Oncology

## Abstract

To investigate the performance of multiparametric ultrasound for the evaluation of treatment response in breast cancer patients undergoing neoadjuvant chemotherapy (NAC). The IRB approved this prospective study. Breast cancer patients who were scheduled to undergo NAC were invited to participate in this study. Changes in tumour echogenicity, stiffness, maximum diameter, vascularity and integrated backscatter coefficient (IBC) were assessed prior to treatment and 7 days after four consecutive NAC cycles. Residual malignant cell (RMC) measurement at surgery was considered as standard of reference. RMC < 30% was considered a good response and > 70% a poor response. The correlation coefficients of these parameters were compared with RMC from post-operative histology. Linear Discriminant Analysis (LDA), cross-validation and Receiver Operating Characteristic curve (ROC) analysis were performed. Thirty patients (mean age 56.4 year) with 42 lesions were included. There was a significant correlation between RMC and echogenicity and tumour diameter after the 3rd course of NAC and average stiffness after the 2nd course. The correlation coefficient for IBC and echogenicity calculated after the first four doses of NAC were 0.27, 0.35, 0.41 and 0.30, respectively. Multivariate analysis of the echogenicity and stiffness after the third NAC revealed a sensitivity of 82%, specificity of 90%, PPV = 75%, NPV = 93%, accuracy = 88% and AUC of 0.88 for non-responding tumours (RMC > 70%). High tumour stiffness and persistent hypoechogenicity after the third NAC course allowed to accurately predict a group of non-responding tumours. A correlation between echogenicity and IBC was demonstrated as well.

## Introduction

Early and accurate prediction of tumour response to neoadjuvant chemotherapy (NAC) in breast cancer patients is crucial and may guide clinical decisions, including surgical means such as mastectomy or breast conserving surgery (BCS), partial breast irradiation, or axillary dissection. The current radiological methods provide a challenge for radiologists when making such decisions.

The usefulness of various diagnostic methods, including physical examination, mammography (MMG), ultrasound (US), magnetic resonance imaging (MRI) and positron emission tomography/computed tomography (PET/CT) has been examined^1^. There are currently no standard diagnostic criteria or reference methods for monitoring the effectiveness of NAC. Further, few preliminary studies have shown promising results using enhanced contrast magnetic resonance imaging (CE-MRI), diffusion weighted magnetic resonance imaging (DW-MRI), or positron emission tomography/computed tomography (PET/CT), revealing the need for further research. Predicting early tumour responses and development of chemoswitch strategies based on ultrasound, would provide an advantage over other imaging methods based on the non-invasive and easily available characteristics.

Tumour stiffness and vascularity could be assessed by US^2–6^. Ma et al. published that both sonoelastography techniques, strain elastography (SE) and shear wave elastography (SWE), exhibited similar performances for predicting the response to NAC^7^. Previous studies have shown that contrast enhanced ultrasound (CEUS) in combination with SE [using the Strain Ratio (SR)] is very accurate in assessing the effectiveness of NAC in breast cancer^8^. Moreover, previous studies have shown promising results in assessing breast tumors using combining B-mode US features and quantitative parameters such as CEUS, Doppler, and elastography^9–11^. This multi-parameter approach is particularly important in the assessment of post-NAC response, as it asseses tissue structural changes, changes in angiogenesis, and tumor biomechanical properties in the context of histopathological verification.

Another promising direction for the early evaluation of tumour responses to NAC therapy is the use of quantitative ultrasound (QUS). Some initial studies were able to identify changes on QUS as early as one week after NAC, using parameters such as integrated backscattering coefficient (IBC) and average acoustic concentration^12,13^.

In the present study, we examined IBC and the echogenicity of ultrasound images of tumours undergoing therapy. Echogenicity depends on the amplitude of backscattered waves. IBC is a quantitative measure of the effectiveness of ultrasonic backscatter. Thus, both parameters are related to the amount of scattered ultrasonic energy. IBC is determined from raw Radio Frequency (RF) signals, and it must be emphasised that this quantitative technique is operator-independent. Echogenicity of the tumours is assessed on the basis of B-mode images, in accordance with the BIRADS lexicon, and in correlation with subcutaneous fat, although this is qualitative. Changes in IBC values are associated with the modification of specific tumour tissue structures^12^. We hypothesised that both parameters, echogenicity and IBC are correlated, and tissue changes affecting IBC also cause changes in echogenicity.

We examined the performance of multi-parametric ultrasound metrics, including echogenicity, stiffness, maximal tumour diameter and vascularity to diagnose treatment response in neoadjuvant chemotherapy (NAC) patients with respect to histopathological results of residual malignant cell (RMC). IBC was used to explain the correlation between echogenicity and RMC to better understand its changes during NAC courses.

## Materials and methods

### Patients

This prospective study protocol was approved by the ethics commission of the Maria Skłodowska–Curie Memorial Cancer Centre and Institute of Oncology, Warsaw, Poland, and written informed consent was obtained from each patient. From April 2016 to November 2018, US breast examinations were performed on 30 patients with a total of 42 breast lesions (eight women had bifocal lesions and two had trifocal lesions). All women qualified for NAC at the Oncology Clinic. The inclusion criteria were: maximum diameter of the tumour < 4 cm, multicentre ≤ 3 if in another quadrant or/and breast, immunohistological subtype and lymph nodes status. US examinations were performed before NAC and 7 days after subsequent NAC courses (first to fourth). NAC were administered under the international guidelines, according to the previously detailed protocol^14^. AC (doxorubicin, cyclophosphamide) was used at the beginning (from the first to fourth course), then continuing with taxol. Patients with HER2+ receptor positive tumours were treated with trastusumab with taxol. One patient, with a history of contralateral breast cancer from 5 years prior to the study, was treated with AT (doxorubicin, docetaxel). All patients underwent a mastectomy with lymphadenectomy at the end of the chemotherapy.

### Histology

In all patients, core needle biopsies (CNB), after administration of 2% lidocaine, were performed before NAC treatment. Biopsies were performed using a 14GA diameter biopsy needle (Pro-Mag). Three to five cores were retrieved from each lesion. The same pathologist, with 25 years of experience in oncological pathology, assessed the resected tumours and cores from each CNB. Based on the pathological assessment of the breast tissue from CNB, the grade of malignancy, cancer subtype and immunohistochemistry results were obtained. After operation, information on the tumour responses to treatment, including cellularity (percentage of the RMC), was obtained based on the residual tumour burden assessment^15^.

### Ultrasonic data acquisition and evaluation

US examinations of patients were performed at the Department of Ultrasound, Institute of Fundamental Technological Research, Polish Academy of Sciences in Warsaw. Data acquisition was performed using an US scanner (Ultrasonix Sonix Touch-Research) equipped with a linear array transducer L14-5/38 and the transmit frequency set to 10 MHz. The examinations were carried out according to dedicated US protocol including the assessment of the following parameters from the B-mode image: echogenicity, stiffness, maximal diameter and vascularity of the tumours. The IBC was estimated off-line from acquired raw US data.

The assessment of focal lesions in the breast was based on the guidelines of the American College of Radiology (BI-RADS lexicon) and the standards of the Polish Ultrasound Society^16,17^. US examinations were performed by a single radiologist with 19 years of experience in breast imaging and 8 years of experience in performing SE. Data were recorded from four cross sections of each breast tumour (radial, radial + 45°, anti-radial, anti-radial + 45°).

### Echogenicity of the tumours

Tumour echogenicity (ECHO) was assessed according to ACR BI-RADS, using the grey level of the standard tumour image obtained in B-mode, compared to adipose tissue in pre-glandular zones. One of the four following echogenicity levels was assigned to each tumour image before and after NAC: (1) hypoechoic, (2) hypo- and isoechoic (mixed), (3) isoechoic and (4) hyperechoic.

### Tumour stiffness (sonoelastography)

The SE technique was used according to guidelines from the World Federation for Ultrasound in Medicine and Biology^18^. The tumour stiffness (ELASTO) was quantified using the Tsukuba scale, a 5-point scale of classification, ranging from Tsukuba 1 (strain is seen in the entire lesion) to Tsukuba 5 (no strain is measured in the lesion or surrounding tissue).

### Maximum tumour diameter

The maximum tumour diameter was selected from two tumour sections (radial and anti-radial) according to BI-RADS lexicon for breast ultrasound. For classification, changes in the maximum diameter of the tumour (relative diameter, DIAM-REL) were used to determine the ratio of the maximum diameter of the tumour after subsequent courses of the NAC and the maximum diameter of the tumour before treatment.

### Vascularisation of the tumours

Tumour vascularisation (VASC) was assessed using the colour Doppler technique and the following 3-point scale: (1) lack of vascularity, (2) peripheral vascularity, (3) central and peripheral vascularity.

### Quantitative parameter: integrated backscatter

The IBC was determined from the backscattering coefficient in the frequency range 5–12 MHz, corresponding to the transducer band. The method was described in detail previously^13^. A reference phantom (1126 B, Dansk Phantom Service) was used in the measurements and the RF data was collected from a 3 × 3 mm window, which was a sufficient size to provide reliable values of scattering parameters. A sliding window technique was used with a one-pixel step to obtain parametric maps of the IBC distribution in the tumour^19^. For each tumour, the average IBC value was calculated from all windows of four parametric maps corresponding to the four tumour sections.

### Statistical methods

Statistical analysis was performed using the Matlab (MathWorks, 2018) software package. The correlation analysis included calculation of Pearson and Spearman coefficients. A significance level of p = 0.05 was used for statistical hypothesis testing. For the three individual parameters, including echogenicity, tumor stiffness, and relative tumor diameter, the classification of non-responders versus responders was based on the Linear Discriminant Analysis (LDA) model which is a generalization of Fisher's linear discriminant^20^. The LDA model consisted of identifying hyperplanes (in n-dimensional space, where n is the number of parameters used in the classification model) that best separate tumors with different treatment responses.

The classification performance of each individual parameter was cross-validated using the "Leave-One-Out" technique^21^. The evaluation of the classification results was based on the Receiver Operating Characteristic (ROC) curve and the area under the ROC curve (AUC)^22^. The "optimal" cut-off point of the ROC curve was defined as the point closest to the point (0, 1)^23^. Classification matrices obtained for cut-off points enabled the calculation of sensitivity, specificity, positive predictive value (PPV), negative predictive value (NPV) and accuracy. Confidence intervals were determined for a confidence level of 0.95 using the bootstrap procedure.

In the classification based on two-parameter classifiers, the LDA classification model was also used. Cross-validation was based on the "Leave-One-Out" technique, and ROC curve analyses were used to calculate sensitivity, specificity, positive predictive value (PPV), negative predictive value (NPV) and accuracy.

According to histopathological verification after NAC and surgery, two cut-off values for RMC were considered, RMC = 30% and RMC = 70%. For the RMC = 30% cut-off, the tumours were divided into responding, RT_30,_ and non-responding, N-RT_30_, corresponding to RMC ≤ 30% and RMC > 30%, respectively. Similarly, for the RMC = 70% cut-off, the group of N-RT_70_ tumours had RMC scores ≥ 70%, and RT_70_ tumours had RMC < 70%. The goal of this approach was to identify patients who responded very poorly to therapy, which we assumed would have RMC > 70%, and responded well, i.e. with RMC < 30%.

### Ethical approval

All procedures performed in studies involving human participants were in accordance with the ethical standards of the institutional and/or national research committee and with the 1964 Helsinki declaration and its later amendments or comparable ethical standards. This article does not contain any studies with animals performed by any of the authors.

### Informed consent

Informed consent was obtained from all individual participants included in the study.

## Results

Three patients did not complete the study leading to modification of the NAC data. All remaining patients underwent a full course of NAC therapy. The mean age of the 30 patients (42 breast tumours) was 56.43 years (range 32–83 years; median 54.50 years, SD 14.67). Histopathological verification before surgery revealed invasive carcinoma NST G2 (22 patients), G3 (9 patients) and G1 (11 patients). There were 8 luminal A cancers, 21 were luminal B, 6 were TNBC and 7 tumours were HER2^+^. Clinical details of the patients from the study are shown in Table 1.

**Table 1:** Patient characteristics including histological findings.

Patients	Number of patient	30
	Mean age in years	56.43
	Age range in years	32–83
Tumor histology	Invasive ductal carcinoma (IDC)	42
	IDC with ductal carcinoma in situ	17
Receptor status	Luminal A	8
	Luminal B	21
	TNBC	6
	HER 2+	7
Pathological response (RMC%)	0	10
	< 29	21
	30–69	10
	> 70	11
Surgical treatment	Mastectomy	30

Histopathological examination after final NAC and surgery revealed 21 tumours with 0–29% RMC, including 10 tumours with RMC = 0 (pathological complete response pCR), 10 tumours with RMC of 30–69%, and 11 with RMC > 70%.

No significant correlations were found between RMC and US measurements performed after the first cycle (p ≥ 0.06, Table 2). A significant correlation was found between RMC and the following parameters measured after the third course of NAC: echogenicity, tumour stiffness and relative tumour diameter (Table 2). Additionally, the correlation with tumour stiffness was significant following the 2nd NAC cycle. This provided the basis for using these parameters to classify tumours as responding or not responding. No significant correlation was found between RMC and vascularisation after any of the NAC courses. A significant correlation was found between IBC and echogenicity after the second, third and fourth NAC courses. Results are presented in Table 3.

**Table 2:** The correlation coefficients (Pearson and Spearman) between the RMC and the relative tumour diameter, echogenicity, tumour stiffness and vascularity, p-value is given in brackets.

	Pearson r, (p)				Spearman r, (p)			
	Exam #1	Exam #2	Exam #3	Exam #4	Exam #1	Exam #2	Exam #3	Exam #4
DIAM-REL	0.11 (0.49)	0.14 (0.38)	0.32 (0.04)	0.25 (0.12)	0.09 (0.56)	0.16 (0.30)	0.33 (0.03)	0.25 (0.12)
ECHO	− 0.22 (0.16)	− 0.23 (0.15)	− 0.50 (0.00)	− 0.48 (0.00)	− 0.19 (0.22)	− 0.26 (0.10)	− 0.49 (0.00)	− 0.45 (0.00)
STIF	0.29 (0.06)	0.52 (0.00)	0.50 (0.00)	0.52 (0.00)	0.30 (0.05)	0.42 (0.01)	0.50 (0.00)	0.43 (0.01)
VASC	0.00 (1.00)	0.17 (0.30)	0.29 (0.06)	0.14 (0.39)	0.02 (0.90)	0.21 (0.19)	0.30 (0.06)	0.22 (0.18)

**Table 3:** Correlation between echogenicity and IBC after subsequent NAC courses.

	NAC 1	NAC 2	NAC 3	NAC 4
Correlation	0.27	0.35	0.41	0.30
*p*-value	0.1	0.01	0.004	0.003

**Figure 1:**
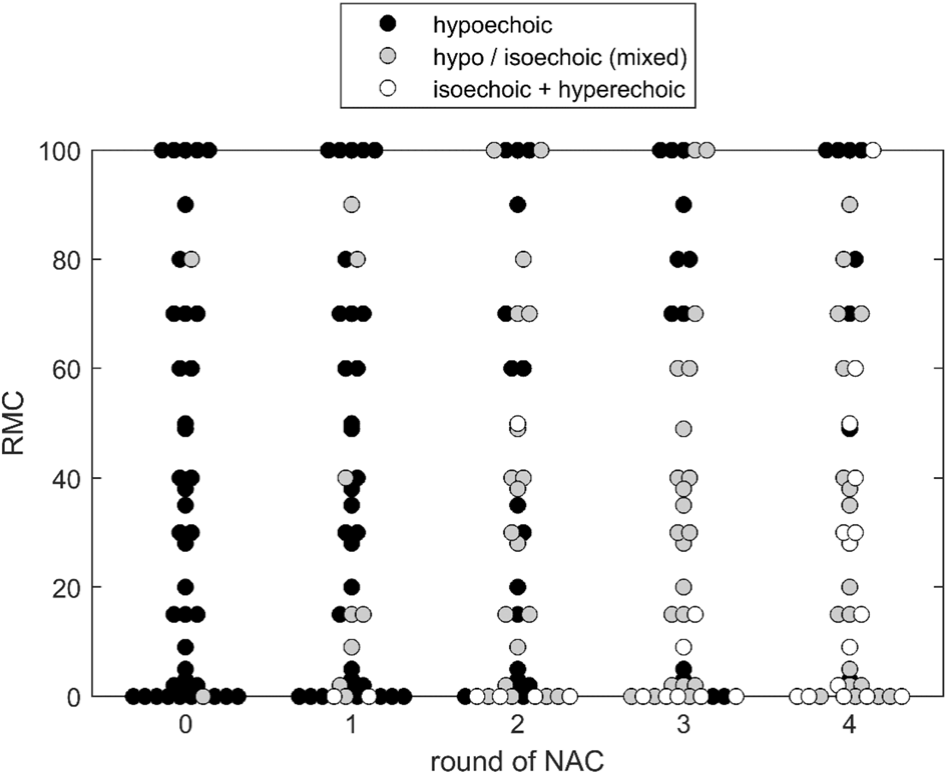
The RMC (%) value of tumour in relation to tumour echogenicity after subsequent courses of NAC. Measurements were determined before the treatment (0) and after subsequent NAC courses (from 1 to 4). Isoechoic and hyperechoic tumours (echogenicity levels 3 and 4) are presented together due to low number of the latter. (One case of hyperechoic tumour was observed after the 3rd and two after the 4th NAC course).

Before treatment, 40 lesions were hypoechoic in B-mode US measurement (average RMC = 36%) and 2 presented with mixed echogenicity (hypo- and isoechoic, average RMC = 40%) compared to fat tissue. After the third course of NAC, 12 tumours, with an average RMC value of 58%, were persistently hypoechoic in grey-scale US. In iso- and/or hyper-echoic tumours, the average RMC value after three NAC courses was 3% (n = 7). The results are presented in Fig. 1.

**Figure 2:**
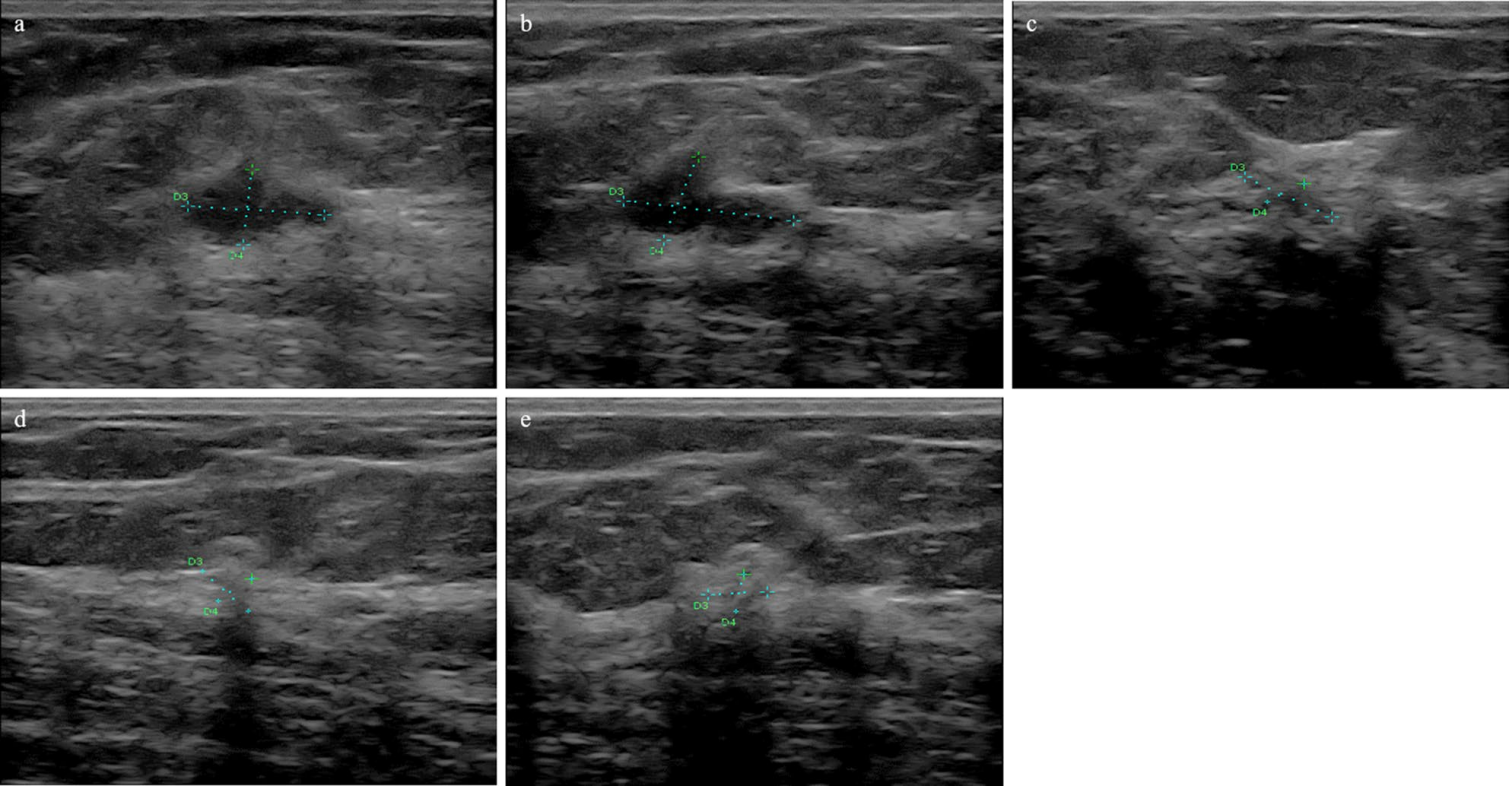
Ultrasound results from a 56-year-old patient with breast cancer (NST Grade 3, TNBC, Ki 67–30%). In B-mode examination (**a**) before treatment, the tumour was hypoechoic. After the first (**b**), second (**c**), third (**d**), and fourth (**e**) course of the NAC, the echogenicity increased (histopathological verification: RT, RMC = 0).

**Figure 3:**
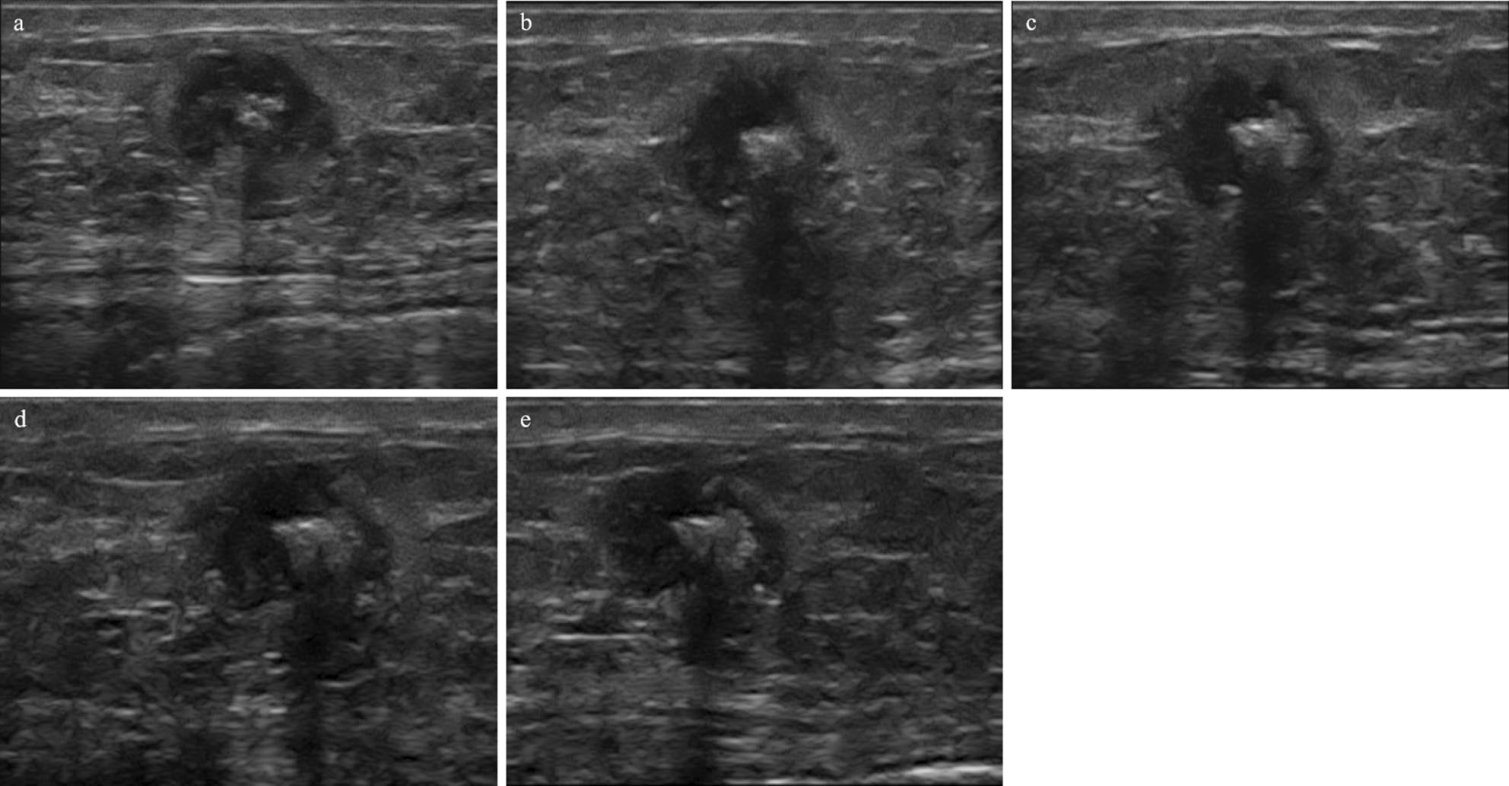
Ultrasound results from a 65-year-old patient with breast cancer (carcinoma invasivum apocrinale, Grade 3, TNBC, Ki 67–20%). In B-mode examination before treatment (**a**) the tumour was hypoechoic with calcification. After the first (**b**), second (**c**), third (**d**), and fourth (**e**) course of the NAC, echogenicity remains unaltered (histopathological verification: N-RT, RMC = 100%).

Examples of B-mode images for the case N-RT (RMC = 100%) and RT (RMC = 0) are shown in Figs. 2 and 3, respectively.

**Figure 4:**
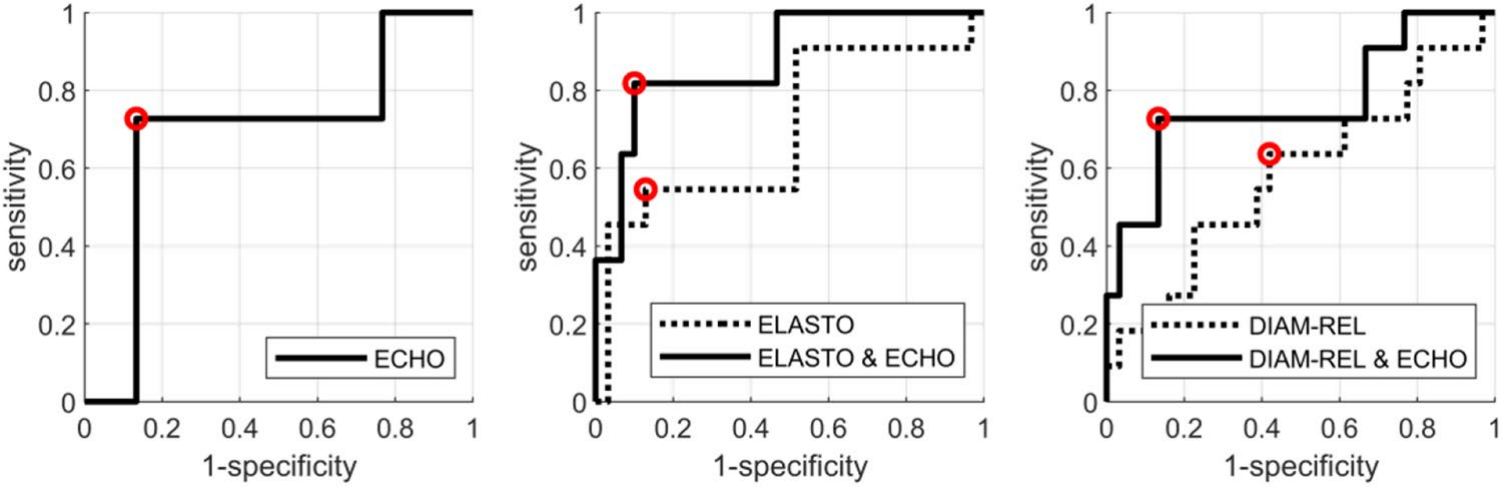
ROC curves for classification of non-responding tumours (RMC ≥ 70%) after the 3rd NAC course. The classifiers were based on ECHO (left), ELASTO alone and with ECHO (middle), and DIAM-REL alone and with ECHO (right). Red markers indicate the operating points for which the parameters are shown in Table 4.

**Figure 5:**
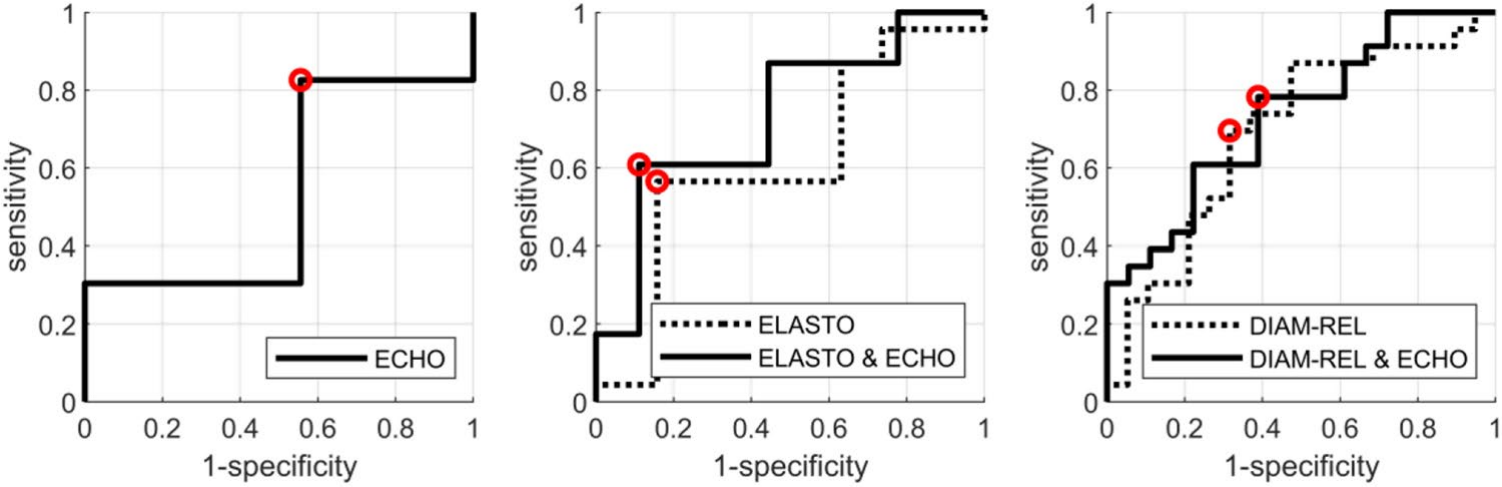
ROC curves for classification of responding tumours (RMC ≤ 30%) after the 3rd NAC course. The classifiers were based on ECHO (left), ELASTO alone and with ECHO (middle), and DIAM-REL alone and with ECHO (right). Red markers indicate the operating points for which the parameters are shown in Table 5.

Univariate and multivariate analyses of classification efficiency expressed by Receiver Operating Characteristic (ROC) curve for echogenicity, stiffness and relative diameter of tumour were performed (Figs. 4, 5).

The best results were observed upon the combination of two parameters: echogenicity and elasticity, as determined after the third NAC course, and relative diameter and echogenicity. Classification results based on these predictors are shown in Tables 4 and 5 for both cut-off values. Predictions based on combined echogenicity and stiffness parameters for a 70% cut-off after three NAC courses achieved 82% sensitivity, 90% specificity, PPV 75%, NPV 93%, and 88% accuracy. There were only two false positive and three false negative cases. The corresponding statistical parameters for the 30% RMC cut-off value were 61%, 89%, 88%, 64% and 73%, respectively.

**Table 4:** Evaluation of selected classifiers of non-responding tumours (RMC ≥ 70%) after third NAC course. Evaluation parameters are given with the 95% confidence level (95% CL).

	AUC (CI)	sens (CI)	spec (CI)	acc	PPV	NPV
ECHO	0.69 (0.48–0.88)	0.73 (0.45–0.91)	0.87 (0.73–0.97)	0.83 (0.71–0.93)	0.67 (0.45–0.90)	0.90 (0.80–0.97)
ELASTO	0.70 (0.48–0.88)	0.55 (0.27–0.82)	0.87 (0.73–0.97)	0.78 (0.66–0.90)	0.60 (0.33–0.88)	0.84 (0.75–0.93)
ELASTO and ECHO	0.88 (0.72–0.97)	0.82 (0.55–1.00)	0.90 (0.77–1.00)	0.88 (0.76–0.95)	0.75 (0.54–1.00)	0.93 (0.84–1.00)
DIAM-REL	0.59 (0.37–0.79)	0.64 (0.36–0.91)	0.60 (0.43–0.77)	0.61 (0.46–0.76)	0.37 (0.22–0.53)	0.82 (0.69–0.95)
DIAM-REL and ECHO	0.77 (0.55–0.92)	0.73 (0.45–0.91)	0.87 (0.73–0.97)	0.83 (0.71–0.93)	0.67 (0.45–0.90)	0.90 (0.80–0.97)

*Sens *sensitivity, *spec *specificity, *acc *accuracy, *PPV *positive predictive value, *NPV *negative predictive value.

**Table 5:** Evaluation of selected classifiers of responding tumours (RMC ≤ 30%) after third NAC course. Evaluation parameters are given with the 95% confidence level (95% CL).

	AUC (CI)	sens (CI)	spec (CI)	acc	PPV	NPV
ECHO	0.54 (0.34–0.72)	0.83 (0.65–0.96)	0.44 (0.22–0.67)	0.66 (0.51–0.78)	0.66 (0.56–0.77)	0.67 (0.42–0.90)
ELASTO	0.62 (0.43–0.79)	0.57 (0.35–0.74)	0.83 (0.61–1.00)	0.68 (0.54–0.80)	0.81 (0.63–1.00)	0.60 (0.48–0.74)
ELASTO and ECHO	0.73 (0.56–0.87)	0.61 (0.39–0.78)	0.89 (0.72–1.00)	0.73 (0.59–0.85)	0.88 (0.71–1.00)	0.64 (0.52–0.77)
DIAM-REL	0.68 (0.50–0.84)	0.70 (0.48–0.87)	0.67 (0.44–0.89)	0.68 (0.54–0.80)	0.73 (0.59–0.88)	0.63 (0.47–0.81)
DIAM-REL and ECHO	0.73 (0.56–0.87)	0.78 (0.61–0.91)	0.61 (0.39–0.83)	0.71 (0.56–0.83)	0.72 (0.60–0.86)	0.69 (0.50–0.88)

*Sens *sensitivity, *spec *specificity, *acc *accuracy, *PPV *positive predictive value, *NPV *negative predictive value.

## Discussion

In this study, we used a multi-parameter approach based on changes in tumour echogenicity, stiffness, maximum diameter and vascularity to indicate response to NAC. We found that tumour stiffness decreases significantly in tumours which respond to NAC as early as the second cycle. Similar results were published by Fernandes et al., who monitored 92 locally advanced breast cancer using SR. The authors reported that tumors without pCR were observed to decrease in SR (by 3%), whereas ones with pCR were observed to significantly decrease in SR (by 12%), after the second week of treatment^6^.

We have also found differences in echogenicity and diameter variation between responding and non-responding tumours can be detected after the third NAC cycle. On the other hand, vascular changes do not give sufficient information to predict response to treatment.

Persistent tumour hypoechogenicity and high stiffness after the third NAC cycle allowed accurate prediction of the N-RT_70_ group. After the third dose of chemotherapy, echogenicity of the tumour, used as a single parameter, enabled identification of non-responding tumours with a sensitivity of 73% and a specificity of 87%. The addition of tumour stiffness to the classification score increased sensitivity and specificity to 82% and 90%, respectively. These findings associated with the echogenicity changes are in line with our previous preliminary research on 19 tumours^24^. The absence of a change in echogenicity (persistence of hypoechogenicity) after the third NAC course was the most accurate parameter for predicting a poor response to NAC, with a high PPV of 92.31%, NPV of 83.33%, and a cut-off for RMC of 30%. In a recent study for echogenicity PPV was 67%, NPV was 67%, however, in our study the NPV was higher with a cut-off value of 70%, and equalled 90%.

In this study, we assessed the correlation between IBC and echogenicity and found it to be statistically significant. When determining the IBC coefficient, all frequencies in the considered band are given the same weight. Echogenicity, however, depends on the amplitude of the backscattered transmission signal, and most of the energy is concentrated around the central frequency of the transducer bandwidth. It can, therefore, be assumed that the absence of any change in IBC is equivalent to no change in echogenicity, whereas a change in IBC does not always indicate a change in echogenicity. In addition, IBC is determined from RF signals, and echogenicity is assessed based on B-mode images. The B-mode image is calculated from the processed RF signal amplitude distribution by applying logarithmic compression. Such processing leads to an equalization of the image brightness and causes, among other effects, a reduction in the brightness of high-amplitude echoes, which has a significant impact on the value of IBC. Consequently, the B-mode imaging provides information that is analogous, but not equal, to qualitative tissue echogenicity.

The increase in the echogenicity of tumours, in the case of a positive response to NAC, can be explained by analysing changes within the cancer cells and stroma following subsequent courses of chemotherapy. Various pathological cellular changes have been described for tumours showing complete response within the first few weeks after chemotherapy. In tumour cells, the most striking manifestation of the treatment is a decrease in the tumour cellularity and fragmentation of the cell nucleus^25^. Nuclear fragmentation is associated with cell death and leads to an increase in the concentration of acoustic scatterers. However, the IBC depends on the size of the scatterers rather than the concentration^26^. After 1–2 months, the stroma collagenisation, formation of excess fibrous connective tissue, and microcalcifications have been observed in tumours that respond to the treatment, and these changes lead to increased acoustic impedance of tissue^25^. This effect increases ultrasound reflection and can be seen as an increase in the echogenicity of the tumour image. Based on histological specimen, it has been shown that isoechoic structures in the breast are representative of stroma with fibrous connective tissue^27^.

In the case of resistant tumours, no reduction of malignant cells is observed and only some cancer cells tend to grow with cytoplasmic vacuoles on microscopic examination^25^. No significant or minor changes occur in the stroma. In our study, we demonstrated that hypoechogenicity in the B-mode image correlates with high RMC. As an example, in N-RT_70_, low echogenicity remains unchanged in 67% of tumours after 3 courses of NAC.

Matsuda et al., in a study of 52 TNBC tumours, measured the changes in the brightness of the ultrasound images of treated tumours. They observed that quantification of echogenic changes can predict the clinical response^28^. The authors reported 73.7% and 81.8% sensitivity and specificity, respectively. However, their research was considerably different from ours. They used tumour echogenicity data before and after NAC treatment, whereas, in our study, we relied entirely on data after the third NAC cycle. Further, in our study only 6/42 (14%) of the tumours were TNBC. It has been reported that some aggressive forms of breast cancer, such as TNBC and HER2+, achieve over 50% pCR in NAC therapy, which presents a significant difference between the studied cohorts.

Our study has shown that the combined measurement of alteration in maximum diameter and echogenicity (AUC 0.77) is a better predictor than maximum diameter alone (AUC 0.58, sensitivity of 70%, specificity of 68%) for N-RT_30_. A similar result regarding the size of tumours was documented by Baumgartner et al.^29^. On the basis of 124 breast cancers, their data indicated that ultrasound does not predict pCR with sufficient accuracy, their methods achieved 60.8% sensitivity and 78% specificity.

In a study involving 832 patients, Marinowich et al. estimated the accuracy of using US determined tumour size following the second cycle of NAC for pCR prediction based on RECIST and WHO criteria^30^. This study also resulted in relatively low pCR identification, although with a greater sensitivity of 77.4% but lower specificity of 50.8%.

It should be emphasised that two-parameter evaluation based on echogenicity and tumour stiffness or diameter can easily be applied in clinical practice. We believe that it is a potentially valuable tool for monitoring NAC therapy, with a wide range of applications, in particular for predicting N-RT.

Finally, the limitations of the present study should be discussed. All breast cancer molecular subtypes were assessed together. It should also be noted that the assessment of tumour echogenicity is a qualitative assessment and depends on the experience of the physician. Therefore, confirmation of our results by other research groups would be very valuable. Further research is needed using larger tumour cohorts broken down into molecular subtypes. This type of study design may provide differing results from the present study which did not separate tumours based on molecular characteristics. Another limitation of our study was the lack of iso- and/or hyper-echoic tumours before the treatment. In the future, we would like to continue the US multi-parameter study, using SWE to evaluate stiffness and compare the method’s effectiveness in various biological subtypes of breast cancer.

## Conclusion

This study shows that evaluating two tumours associated ultrasound parameters together, echogenicity and stiffness, during NAC therapy are the best predictors of tumour response to chemotherapy. High tumour stiffness and persistent hypoechogenicity after the third NAC cycle allowed for accurate prediction of non-responding tumours (N-RT_70_). For tumours, which respond well to therapy, the combination of tumour diameter changes with echogenicity also allowed for improved classification of the tumours after the third dose of NAC. Analysis of IBC coefficient changes allowed the researchers to link changes in tumour echogenicity on B-mode images with changes in tumour tissue during NAC therapy.
